# Effect of Waterglass on the Hydration Process of Slag-Fly Ash-Based Geopolymer

**DOI:** 10.3390/ma18112450

**Published:** 2025-05-23

**Authors:** Ran Hai, Qingpu Guan, Xiaorong Zhang, Fei Yang, Li Cui, Junxia Liu

**Affiliations:** 1 School of Materials Science and Engineering, Henan University of Science and Technology, Luoyang 471023, China; lilyhai_2001@163.com; 2 School of Intelligent Construction and Civil Engineering, Zhongyuan University of Technology, Zhengzhou 450007, China; guanqingpu2233@163.com (Q.G.); 18839339439@163.com (X.Z.); 3 School of Civil Engineering and Architecture, Henan University of Science and Technology, Luoyang 471000, China; cuili@haust.edu.cn

**Keywords:** geopolymer mortar, waterglass, mechanical properties, hydration heat, hydration products

## Abstract

Geopolymers possess good mechanical properties and durability, and their partial replacement of traditional Portland cement is noteworthy for promoting the development of low-carbon building materials. To clarify the influence mechanism of the mechanical properties of slag-fly ash-based geopolymer mortar, this paper investigated the hydration heat, composition, and morphology of hydration products with various contents and moduli of waterglass. The results showed that the compressive strength of geopolymer mortar increased with increasing waterglass content, and first rose and then fell as the waterglass modulus raised, while its flexural strength increased and then decreased with the growth in both. The compressive and flexural strength of geopolymer mortar with 1.2-modulus waterglass at 20 wt% cured for 28 days were 88.4 MPa and 9.0 MPa, respectively. The hydration temperature and cumulative hydration heat of geopolymer mortar was elevated with the increase in waterglass content, and declined with the rising waterglass modulus. The hydration products of the geopolymer consisted of dense amorphous and flocculent structures wrapped around each other. The microstructure of the geopolymer cured for 3 days was loose when the content of 1.4-modulus waterglass was 5 wt%. The relative areas of the flocculation in the geopolymer cured for 28 days increased while the waterglass modulus was greater than 1.4, forming an interface with the dense amorphous structure generated during the early hydration stage, leading to a decrease in its mechanical properties. Therefore, it is recommended for slag-fly ash geopolymer mortar that the waterglass modulus is between 1.2 and 1.4 and its content is no less than 10 wt% to ensure suitable mechanical properties. This study also provided a referenceable time period for the pouring and operation of the engineering application of slag-fly ash-based geopolymer repair mortar.

## 1. Introduction

Global carbon dioxide emissions from cement production grow at a rate of 6% per year; geopolymer materials have emerged as the answer [1]. Geopolymers are a cementitious material with the Si-O-Al tetrahedral three-dimensional network structure formed by aluminosilicate compounds as a precursor activated by an alkaline activator [2]. Solid wastes, such as fly ash, granulated blast furnace slag, red mud, and construction waste powder, are employed as raw materials to produce geopolymers, achieving better environmental and economic benefits [3,4].

The hydration process and mechanical properties of geopolymers are closely related to its mixture, i.e., types and proportions of activated silica–aluminum powders, water–cement ratio, and activator properties. Metakaolin was the first raw material used for geopolymers because of its excellent volcanic ash properties, but its application is constrained by high energy consumption and high costs during its production process [5,6]. The compressive strength of a slag-based geopolymer reached 18.9 MPa after curing for 2 days, producing a larger linear shrinkage [7]. The content of CaO in fly ash was relatively low, resulting in its lower reactivity at room temperature [8]. Moreover, the addition of 10 wt% slag shortened the initial setting time by 44.5% [9]. Compared with geopolymers derived from a single solid waste, slag-fly ash-based geopolymers exhibit superior condensation time and mechanical properties [10,11].

Furthermore, activator properties, such as acidity or alkalinity, species, composition, and dosage, significantly influence the ‘dissolution-polycondensation’ process of geopolymers [12]. Currently, waterglass is the most widely studied and applied of all strong alkaline activators. Lyu et al. [13] concluded that the compressive strength of geopolymer continues to increase with a decrease in waterglass modulus. Wang et al. [14] concluded that the modulus of waterglass was inversely proportional to the compressive strength in the range of 1.2 to 2.4. However, when the waterglass modulus increased from 1.0 to 1.25, the compressive strength of the geopolymer cured for 28 days increased by 20%, while the curing and hardening time was reduced by 50% [15]. With the increase in the waterglass modulus, the decrease in the alkalinity of the solution slowed down the excitation of the alkali-active components and then delayed the setting of the geopolymer at a macroscopic level [16].

Waterglass provides soluble oligomeric silicate ions for the hydration reaction of geopolymers [17]. When the alkalinity of waterglass was kept at a lower level, limiting the excitation effect of alkali-activated powders, the oligomeric [SiO_4_^4−^] ions in waterglass reacted with free Si^4+^ and Al^3+^ enriched in a fresh mixture to form geopolymer gel [18]. Additionally, variations in waterglass content significantly impact geopolymer properties. An increase in waterglass content makes the solidification and hardening of slag-fly ash-based aggregates faster [19], but its strength increases and then decreases with the increase in the content, and the optimal content is 20% [20].

In summary, the modulus and content of waterglass exert a profound influence on the setting time and mechanical properties of geopolymers, and its influence mechanism is worthy of in-depth exploration. Based on previous research [21], granulated blast furnace slag and fly ash were employed as precursors to preparing geopolymer mortar. By analyzing the composition and microstructure of the hydration products of slag-fly ash-based geopolymers cured for various ages, this study clarified its hydration process. Furthermore, this paper systematically investigated the influence mechanism of waterglass modulus and content on the mechanical properties, hydration temperature, and heat of geopolymer mortar. Eventually, these data and basic theory will contribute to improving the performance of geopolymers and promoting their practical applications.

## 2. Experimental Raw Materials and Methods

### 2.1. Raw Materials

**Figure 1:**
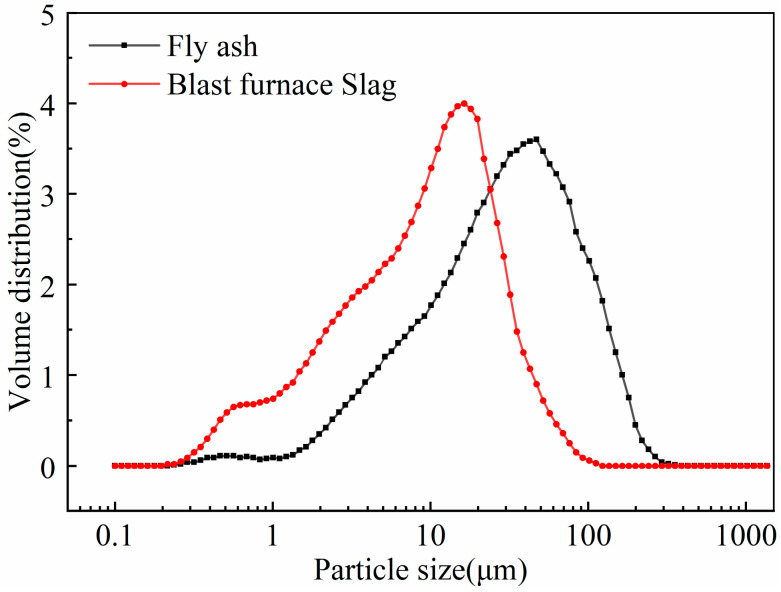
Particle size distribution of fly ash and granulated blast furnace slag.

Grade 2 fly ash and S105 granulated blast furnace slag were employed as an alkali-active precursor of the geopolymer—their particle sizes are shown in Figure 1 and their chemical compositions are listed in Table 1. The waterglass solution had a Baume degree of 40, and its modulus and content were 3.2 and 34 wt%, respectively. The purity of sodium hydroxide was 97%, and was used to adjust the modulus of waterglass. The particle size range of quartz sand was 0.40–0.70 mm.

### 2.2. Mixture of Geopolymer Mortar

In accordance with the results of prior research [21], alkali-active precursors of geopolymer cement consisted of 70% slag and 30% fly ash. Mixtures of the geopolymer mortar are listed in Table 2, wherein the designed waterglass modulus was obtained by adjusting commercially available high modulus waterglass with NaOH according to Equation (1). The specific adjustment results are given in Table 3.

(1) $${\mathrm{Na}}_{2}O\cdot 3.2\;\mathrm{Si}O_{2}+2\mathrm{NaOH}\to {\mathrm{Na}}_{2}O\cdot n\;\mathrm{Si}O_{2}+H_{2}O$$

The geopolymer mortar specimens (40 mm × 40 mm × 160 mm) were vibration-molded in accordance with the Chinese standard GB/T 17671-2021 “Test Method for Strength of Cement Mortar” [22], and there were three samples in each group. After the initial setting of the fresh mixture, the surface was covered with a plastic film. Then, the specimens were cured for 24 h, then demolded and rewrapped with plastic film. Subsequently, the specimens were placed in a standard curing box with a temperature of 20 ± 2 °C and a relative humidity of not less than 95% until the specified curing age was reached. The compressive strength and flexural strength were tested according to GB/T 17671-2021 [22].

### 2.3. Hydration Heat

**Figure 2:**
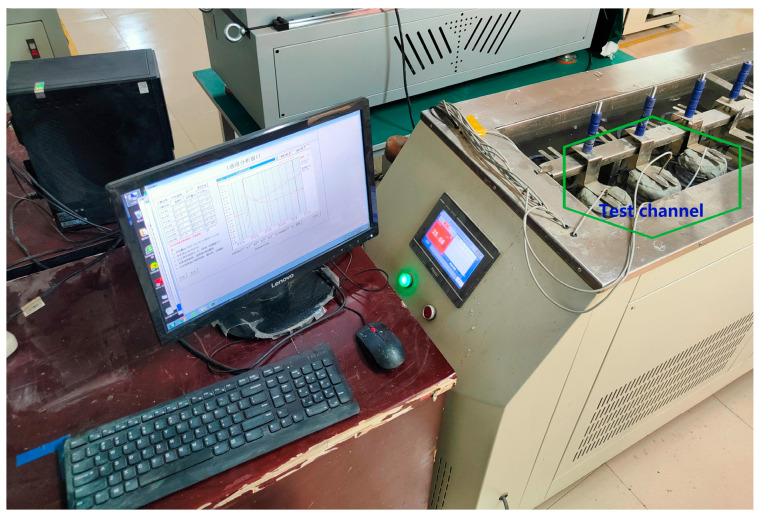
Twelve-channel microcalorimeter test site.

The hydration heat of the geopolymer mortar was tested using a twelve-channel microcalorimeter (as shown in Figure 2) according to the Chinese specification GB/T 12959-2008 “Test Method for Heat of Hydration of Cement” [23]. A total of 1600 g fresh mortar was prepared according to Table 2 prior to testing, and then 800 g of that was put into the test channel, i.e., the insulated wide-mouth bottle. In a constant temperature environment, the calorimeter directly measured the temperature changes in the geopolymer mortar inside the calorimeter caused by its hydration. The tests were repeated three times for each group of geopolymer mortar to screen out the most representative hydration heat curve. The total hydration heat accumulated and dissipated in the calorimeter was calculated by Formula (2).

(2) $$Q_{x}=C_{p}\left(t_{x}-t_{0}\right)-K\sum F_{0-x}$$

where $Q_{x}$ represents the total hydration heat of cement curing for x hours, J. *C_p_* is the total heat capacity of the calorimeter, J/°C. $t_{x}$ is the temperature of the mortar at the corresponding age, °C. $t_{0}$ is the initial temperature of the mortar, °C. *K* is the heat dissipation constant of the calorimeter, J/(°C·h). $\sum F_{0-x}$ is the area between the temperature curve of the mortar curing age from 0 to x and the constant temperature line of the water tank, J.

### 2.4. Analysis of Phase Composition and Microstructure of Hydration Products

The geopolymer cement paste specimens were cured until the predetermined curing age. Before the microscopic tests, the specimens were dried in a vacuum drying oven at 60 °C to a constant weight. Before the Scanning Electron Microscopy (SEM) test, gold sputtering treatment was performed to form a conductive film on the surface of specimens, which was carried out by sputtering Pt powder for 2 min. The morphological characteristics of the hydration products of the geopolymer cement at different ages were observed by a Regulus 8100 electron microscope, Hitachi, Ltd., Tokyo, Japan under the conditions of an accelerating voltage of 5 kV and three spot sizes.

The samples for X-ray diffraction (XRD) analysis were first ground and then sieved through a 75 μm square-hole sieve. XRD analysis was conducted by the D8 ADVANCE X-ray diffractometer, Bruker AXS GmbH, Karlsruhe, Germany under Cu-Kα radiation with a current of 15 mA/40 kV. The diffraction data were recorded within the angular range of 5–90°, at a step size of 0.02°/2θ·min. Subsequently, the mineral composition of the geopolymer hydration products was analyzed through the software named MDI Jade 6 according to XRD spectra.

## 3. Result and Discussion

### 3.1. Mechanical Properties of Geopolymer Mortar

#### 3.1.1. Effect of Waterglass Content on Mechanical Properties

**Figure 3:**
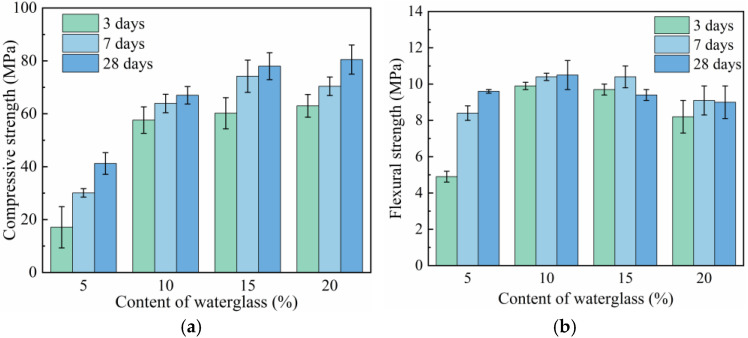
Mechanical properties of geopolymer mortar with different waterglass contents. (**a**) Compressive strength. (**b**) Flexural strength.

The relationship between the waterglass content and the mechanical properties are presented in Figure 3, when the geopolymer mortar was with 1.4-modulus waterglass. Specifically, Figure 3a shows that the compressive strength of the geopolymer mortar cured for various ages all increased with the increase in waterglass content. The compressive strength of the geopolymer mortar cured for 28 days was 41.2 MPa with 5 wt% waterglass, and increased to 60.7 MPa with 10 wt% waterglass. When the waterglass content was 20 wt%, the compressive strength of the geopolymer mortar cured for 3 days and 28 days were 63.0 MPa and 80.5 MPa, respectively, increasing by 268.4% and 95.4% compared with that of 5 wt% waterglass specimens. As can be seen in Figure 3b, the flexural strength of the geopolymer mortar at all curing ages increased first and then declined with the increase in waterglass content. For the specimens with 15 wt% and 20 wt% waterglass, the flexural strength of the geopolymer mortar cured for 28 days was slightly lower than that of at 7 days, experiencing a reduction in strength.

The concentration of [SiO_4_^4−^] and OH^−^ ions contained in the solution of fresh geopolymer mortar was low, when the waterglass content was 5 wt%. Thereafter, the depolymerization of the active silica–alumina precursor reduced accordingly, resulting in the lower strength of geopolymer mortar at all curing ages. With the increase in waterglass content, the poorly polymerized [SiO_4_^4−^] subsequently increased to promote the geopolymer reaction, and it reacted with Ca^2+^ existing in the precursor to form C-S-H gel, all of which were beneficial to the mechanical properties of the geopolymer mortar [24]. As the waterglass content ultimately increased, the concentration of alkali solution in the geopolymer system further elevated with the reduction of free water at the later stages of hydration. This was not conducive to the stability of the C-A-S-H gel in the hydration products, resulting in a slight decrease in the flexural strength of the geopolymer mortar.

#### 3.1.2. Effect of Waterglass Modulus on Mechanical Properties

**Figure 4:**
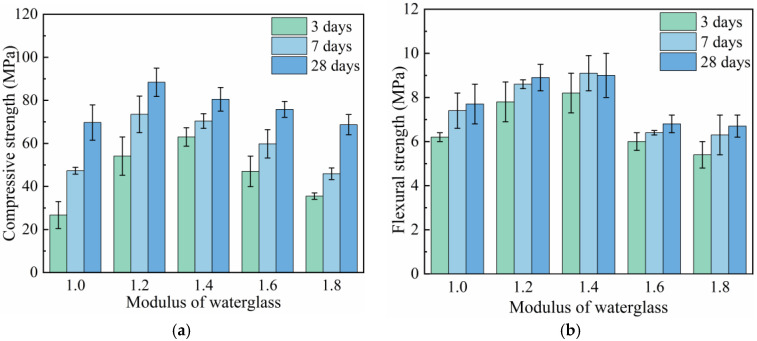
Mechanical properties of geopolymer mortar with different waterglass moduli. (**a**) Compressive strength. (**b**) Flexural strength.

The influence of the waterglass modulus on the mechanical properties of the geopolymer mortar with 20% waterglass is shown in Figure 4. As illustrated in Figure 4a, the compressive strength of the geopolymer mortar at all curing ages exhibited a trend of first increasing and then decreasing with the rising waterglass modulus. The optimum waterglass modulus of the geopolymer mortar cured for 3 days was 1.4, while it was 1.2 at curing ages of 7 days and 28 days. It can be seen in Figure 4b that the flexural strength of the geopolymer mortar first increased and then declined with the increase in waterglass modulus. When the waterglass modulus was 1.4, the flexural strength of the geopolymer mortar cured for 28 days was 9.0 MPa, which was 28.5% higher than that of the specimen activated by 1.0-modulus waterglass. The flexural strength of the geopolymer mortar showed a decreasing trend with the continual increase in the waterglass modulus. When the waterglass modulus was 1.8, the flexural strength of the geopolymer mortar cured for 28 days dropped to 6.1 MPa, which was 32.2% lower than that of the specimen with 1.4-modulus waterglass.

When the waterglass modulus was high, the alkalinity of the geopolymer system was insufficient to dissolve the active SiO_2_ and Al_2_O_3_ enriched in slag and fly ash. Although the content of poor polymerization [SiO_4_^4−^] increased slightly, the weakening of the geopolymer reaction led to a significant decrease in the mechanical properties of the geopolymer mortar. The free OH^−^ in fresh geopolymer increased with the decrease in waterglass modulus, promoting the depolymerization of the silicon–aluminum glass phase enriched in slag and fly ash and the formation of gel deposition. However, when the waterglass modulus of the geopolymer was too low, the high alkalinity promoted the rapid formation of hydration products, which coated the unreacted fly ash and slag particles and hindered the continuation of the geopolymer reaction [25]. In addition, the high alkaline environment was harmful to the stability of the C-S(A)-H gel, deteriorating the mechanical properties of the geopolymer mortar [26].

### 3.2. Hydration Process of Geopolymer Mortar

#### 3.2.1. Effect of Waterglass Content on Hydration Temperature and Hydration Heat

**Figure 5:**
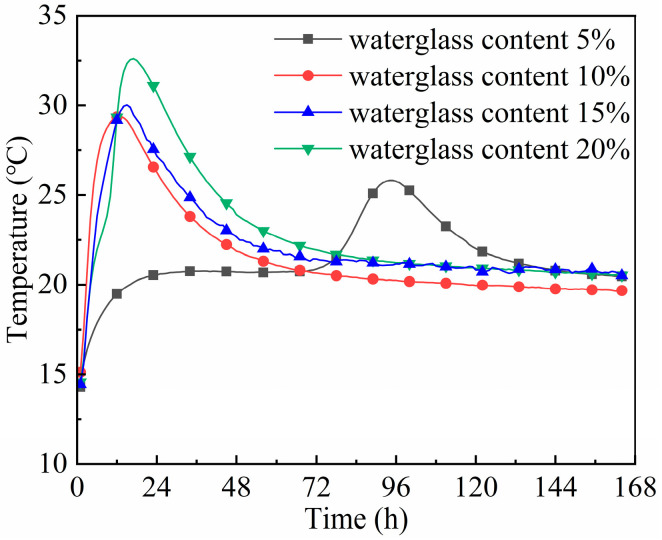
Hydration temperature curve of geopolymer mortar with different waterglass contents.

Figure 5 shows that the peak hydration temperature of the geopolymer mortar was elevated with the increase in waterglass content, and the time taken to reach its peak shortened first and then lengthened. When the geopolymer mortar contained 10 wt% waterglass, its peak hydrated temperature and the time taken were 29.4 °C and 11.6 h, respectively, and they were 32.6 °C and 17.6 h while at 20 wt% waterglass. For the geopolymer mortar with 5 wt% waterglass, its hydration temperature was maintained at about 20 °C in the initial stage, and rose to the peak at 25.9 °C after 84.4 h of hydration.

**Figure 6:**
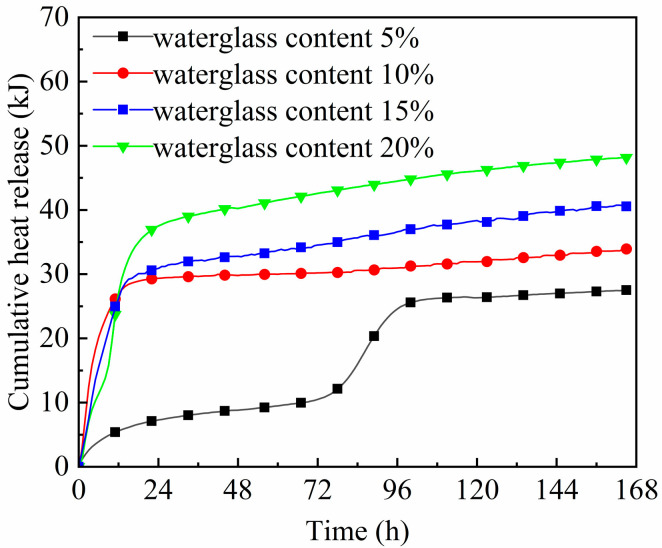
Cumulative hydration heat curve of geopolymer mortar with different waterglass contents.

As is shown in Figure 6, the cumulative hydration heat of the geopolymer mortar increased with the increasing waterglass content. When the waterglass content was 20 wt%, the cumulative hydration heat of the geopolymer mortar reached its maximum, up to 48.2 kJ, and was only 27.5 kJ for the specimen with 5 wt% waterglass. Except for the geopolymer mortar with 5 wt% waterglass, the cumulative hydration heat of the others increased rapidly in its early hydration stage and slowly built up in the later stages. When the waterglass content was 10 wt%, the cumulative hydration heat was 25.3 kJ after 12 h, accounting for 74.6% of its total. When the waterglass content was 15 wt% and 20 wt%, the released hydration heat after 12 h was 65.3% and 56.0% of its total, respectively. This suggested that the increase in the waterglass content improved the later hydration reaction of the geopolymer mortar, which was due to the residual alkali in the geopolymer reacting with slag and fly ash to form C-(A)-S-H gel in the middle and late hydration stages.

The curves of hydration temperatures and heat of geopolymer mortars with 5 wt% waterglass content are of particular interest—Figure 5 and Figure 6. When the waterglass content was 5 wt%, the concentration of [SiO_4_^4−^] and OH^−^ ions in the geopolymer system was too low to maintain its normal ‘dissolution-polycondensation’ reaction [27]. In the early hydration stage, the geopolymer reaction carried on slowly, accompanied by a slow increase in the cumulative hydration heat. After it hydrated for 3 days, the alkali in the geopolymer system reacted with the active SiO_2_ and Al_2_O_3_ enriched in slag and fly ash to form hydrated calcium silicate gel, thus improving its strength. As shown in Figure 3a, the compressive strength of the geopolymer mortar cured for 7 days increased by 76.0% compared with that of cured for 3 days.

#### 3.2.2. Effect of Waterglass Modulus on Hydration Temperature and Hydration Heat

**Figure 7:**
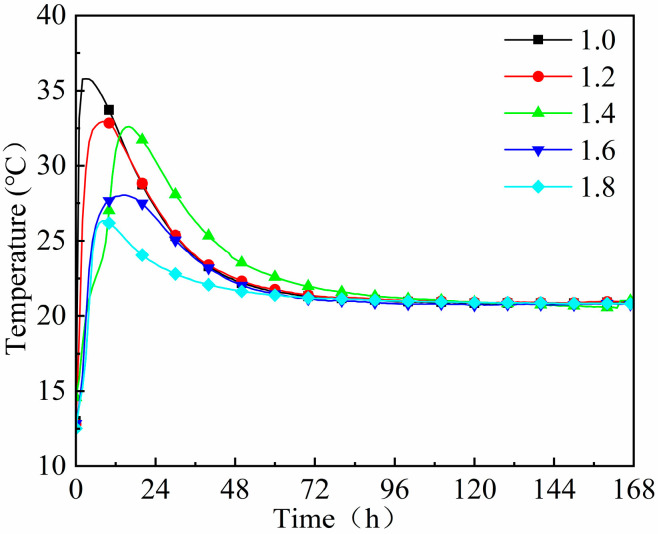
Hydration temperature curve of geopolymer mortar with different moduli of waterglass.

**Figure 8:**
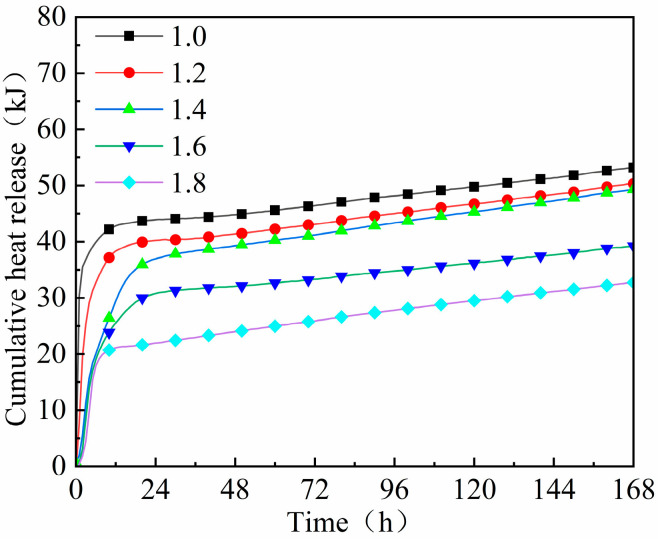
Cumulative hydration heat curve of geopolymer mortar with different moduli of waterglass.

Figure 7 and Figure 8 reveal that the peak hydration temperature and cumulative hydration heat of geopolymer mortar decrease with the increasing modulus of waterglass, while the time taken to reach them was first prolonged and then shortened. When the waterglass modulus was 1.0, the hydration temperature of the geopolymer mortar reached its peak of 35.8 °C in 2.5 h, and its cumulative hydration heat released in 12 h was 42.8 kJ, accounting for 80.6% of its total. In the case of the geopolymer mortar activated by waterglass with the moduli of 1.2, 1.4, 1.6, and 1.8, the time taken to reach their peak hydration temperature was 8.2 h, 17.6 h, 14.3 h, and 8.0 h, respectively, and the cumulative hydration heat released in 12 h accounted for 76.1%, 59.8%, 65.3%, and 64.5% of their total, respectively. The results suggested that the degree and speed of the hydration reaction of the geopolymer mortar were significantly controlled by the waterglass modulus, which should establish an optimum for slag-fly ash-based geopolymer to ensure its condensation performance and volume stability.

The hydration heat of the geopolymer mortar increased sharply in the early stage, and the heat released in 12 h accounted for more than 50% of the total. Subsequently, the overall depolymerization rate slowed down as the geopolymer gels formed and the slurry hardened [28]. The alkalinity of the geopolymer mortar decreased with the increase in waterglass modulus. The low alkalinity was insufficient to fully ‘dissolve’ SiO_2_ and Al_2_O_3_ in the slag and fly ash, and thus reduced the reaction degree of the geopolymer. Therefore, the hydration peak temperature and cumulative heat release were correspondingly reduced. Then, when the geopolymer had a higher waterglass modulus, its macroscopic behaviors showed an obvious decrease in hydration peak temperature, cumulative hydration heat, and mechanical strength. While the waterglass modulus was too low, although the higher alkalinity was beneficial to the geo-polymerization reaction, the reduction in its oligo-silicate also resulted in a decrease in overall hydration products [29].

### 3.3. Composition and Morphology of Hydration Products

#### 3.3.1. Microstructure of Geopolymer with Different Waterglass Content

**Figure 9:**
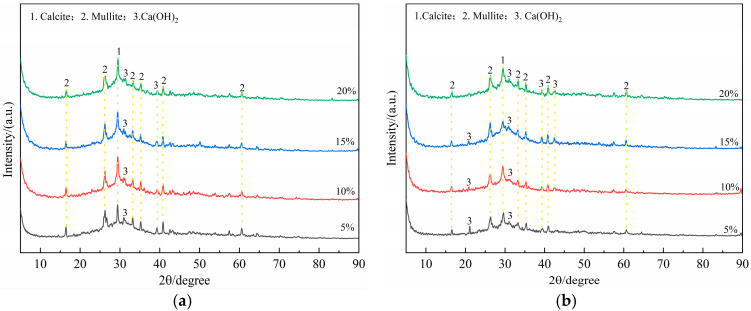
XRD spectrum analysis of geopolymers with various waterglass contents. (**a**) 3 days. (**b**) 28 days.

As can be seen in Figure 9, the characteristic peak of CaCO_3_ was observed in the XRD spectrum of slag-fly ash-based geopolymer cured for 3 days and 28 days. CaCO_3_ was formed by the carbonization of Ca(OH)_2_ during the drying and testing process. The hydration products of geopolymers were mainly composed of amorphous phases, 25°~35° was the characteristic peak of N-A-S-H gel, and 15°~40° was the characteristic peak of C-S-H gel and C-A-S-H gel [30]. Figure 9a exhibits that the height of the N-A-S-H gel peak of the geopolymer cured for 3 days increased slightly with the increase in waterglass content, and the characteristic peak of Ca(OH)_2_ decreased slightly. Figure 9b shows that the intensity of the peak lay in the 15°~40°of XRD spectrum of the geopolymer cured for 28 days and increased with the increasing waterglass content.

**Figure 10:**
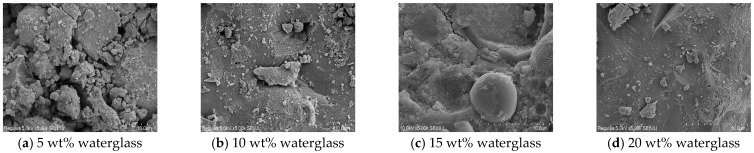
The microstructure of geopolymer with various waterglass contents cured for 3 days.

The micro-morphology of the hydration products of the geopolymer cured for 3 days are presented in Figure 10. From Figure 10a, it was observed that the microstructure of the geopolymer with 5 wt% waterglass was loose. Flocculent hydration products were visible on the surface of parts of the particles and were insufficient at connecting as a whole. Figure 10b,c reveal that a few pores and microcracks were present in the hardened structure of the geopolymer with 10 wt% and 15 wt% waterglass, and the densification was significantly improved compared with that of the specimens with 5 wt% waterglass. Figure 10d showed that the hardened structure of the geopolymer with 20 wt% waterglass was dense and flocculent products were observed only in its localized area.

**Figure 11:**
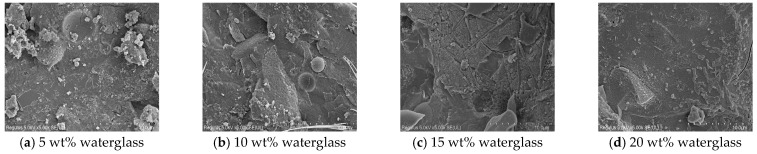
The microstructure of geopolymer with various waterglass contents cured for 28 days.

The morphology of the hydration products of the geopolymer curing for 28 days are shown in Figure 11. The typical geopolymer morphology was observed in the SEM images, whereas the crystalline structure was not found, which was in accordance with the analysis results of the XRD spectrum. As shown in Figure 11a, the compactness of the geopolymer with 5 wt% waterglass was significantly improved compared with the geopolymer cured for 3 days, which led to a significant increase in its mechanical properties at a curing age of 28 days. The SEM images of the hydration products and the micro-compactness in Figure 11b–d showed insignificant differences from the corresponding pictures in Figure 10. Noteworthy is the observation from Figure 11c, that only plate-flaky crystals were interspersed in the amorphous hydration products of the geopolymer.

#### 3.3.2. Microstructure of Geopolymer with Different Waterglass Moduli

**Figure 12:**
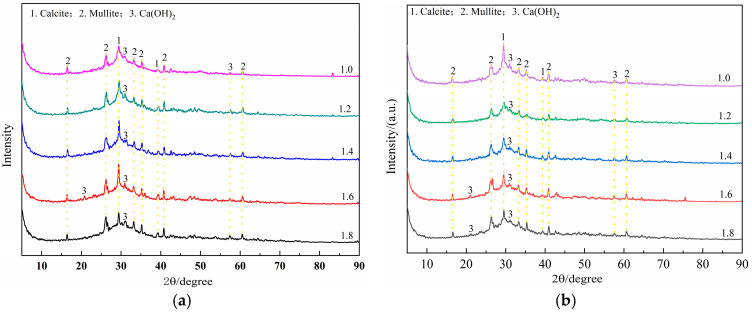
XRD spectra analysis of geopolymers with different moduli of waterglass. (**a**) 3 days. (**b**) 28 days.

Figure 12 indicates that the hydration products of the geopolymer were still dominated by the amorphous phase as the waterglass modulus increased, and no significant difference was observed in comparison with the specimens with various waterglass contents. This suggested that the changes in the waterglass modulus and content did not produce an obvious impact on the types of hydration products at different curing ages, and only had somewhat of an effect on the quantities. While the hydration products of the geopolymer were mainly composed of amorphous N-A-S-H, C-S-H, and C-A-S-H, at the same time, the unreacted slag and fly ash also existed in the form of amorphous SiO_2_ and Al_2_O_3_, thus, the quantitative changes with the waterglass modulus and curing age could not be clearly described by XRD spectra.

**Figure 13:**
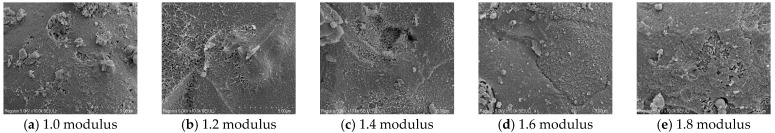
The microstructure of geopolymer with different waterglass moduli cured for 3 days.

Figure 13 shows the morphology of the hydration products of the geopolymer with different waterglass moduli cured for 3 days. As can be seen in Figure 13, the microstructure of the hardened geopolymer consisted of two types of hydration products—one was a dense and compact amorphous product, and the other was a flocculated structure—both of which were wrapped around each other. The relative area of the flocculated product in the observed area enlarged with increasing waterglass modulus, which indicated an increase in the occurrence of alkali-stimulated reactions of fly ash and slag. In the localized areas of Figure 13a–c, prismatic hydration products on the surface of the microfine particles were observed, which were not fully linked to the other products as a whole due to only 3 days of curing. Meanwhile, the continuity of the hydration products shown in Figure 13b,c was relatively high. This is in agreement with the results of the mechanical properties of the geopolymer mortars with different waterglass moduli curing for 3 days. The micromorphology of the geopolymer also explained why no obvious crystals of hydration products were detected in the XRD spectra.

**Figure 14:**
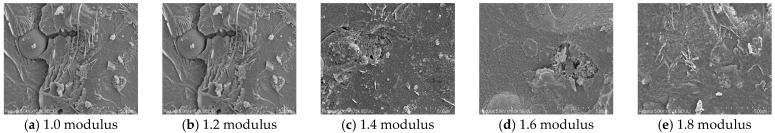
The microstructure of geopolymer with different waterglass moduli cured for 28 days.

The morphology of the hydration products of the geopolymer cured for 28 days is shown in Figure 14. As can be seen in Figure 14a,b, it was observed that layers of tightly packed geopolymers were stacked around the microfine fly ash beads, and almost no other forms of hydration product. When the waterglass modulus was 1.0, the active SiO_2_ and Al_2_O_3_ enriched in slag and fly ash hydrated quickly and produced a temperature elevation after contact with waterglass, which led to a large shrinkage in its later hydration process, and, thus, obvious microcracks were seen in the observation area. In Figure 14c–e, flocculent hydration encapsulated in a layer-stacked geopolymer was observed, suggesting that secondary hydration occurred during the later stages of hydration and produced flocculent C-S-H or C-A-(S)-H as the waterglass modulus increased. The flocculated structures had a relatively lower density and formed a distinct interface with the compact geopolymer structures; both properties were the reason for the corresponding decrease in the mechanical properties of geopolymer mortar with increasing waterglass modulus.

### 3.4. Hydration Mechanism of Geopolymer Cement

#### 3.4.1. Geo-Polymerization Process

Equations (3)–(6) were proposed by Davidovtis [31] to describe the geo-polymerization process. That is, the active silicon–aluminum phase enriched in precursor materials was polymerized into a chain structure by sharing oxygen atoms under the action of a strong alkali. The formation of the geopolymer was divided into two steps. The first step was that fly ash or slag is depolymerized into an oligomeric silicon–aluminum polymer chain in a strong alkaline environment, known as a dissolution process. In the second step, the oligomeric polymer chain was dehydrated and condensed to form a silicon–aluminum polymer chain in a common oxygen atom mode. The negative charge generated by the substitution of the Si monomer by the Al monomer and adsorbed Na^+^, K^+^, and Ca^2+^ thereby maintained the structure’s electric neutrality and formed a stable geopolymer [32].

(3) $$\left({\mathrm{Si}}_{2}O_{5}{,\mathrm{Al}}_{2}O_{3}\right){n+3\mathrm{nH}}_{2}O\underset{\to }{\mathrm{NaOH}}{\;n(\mathrm{OH})}_{3}{-O-\mathrm{Al}}^{-}{\left(\mathrm{OH}\right)}_{3}$$

(4) $${n(\mathrm{OH})}_{3}{-\mathrm{Si}-O-\mathrm{Al}}^{-}{\left(\mathrm{OH}\right)}_{3}\underset{\to }{\mathrm{NaOH}}{{(\mathrm{SiO}-O-\mathrm{Al}}^{-}O-O)}_{n}{+3\mathrm{nH}}_{2}O$$

(5) $${(\mathrm{Si}}_{2}O_{5}{,\mathrm{Al}}_{2}O_{3}{)n+2\mathrm{nSiO}}_{2}{+4\mathrm{nH}}_{2}O\underset{\to }{\mathrm{NaOH}}{n(\mathrm{OH})}_{3}{-\mathrm{Si}-O-\mathrm{Al}}^{-}{\left(\mathrm{OH}\right)}_{2}{-O-\mathrm{Si}(\mathrm{OH})}_{3}$$

(6) $${n(\mathrm{OH})}_{3}{-\mathrm{Si}-O-\mathrm{Al}}^{-}{\left(\mathrm{OH}\right)}_{2}{-O-\mathrm{Si}(\mathrm{OH})}_{3}\overset{\mathrm{NaOH}}{\to }{(-\mathrm{SiO}-O-\mathrm{Al}}^{-}{O-O-\mathrm{SiO}-O-)n+4\mathrm{nH}}_{2}O$$

The geo-polymerization in Equations (3) and (4) reflect the dissolution stage, during which SiO_2_ and Al_2_O_3_ enriched in slag and fly ash dissolved into oligomeric [SiO_4_^4−^] and [AlO_4_^4−^]. Equations (5) and (6) describes the polycondensation progress. Oligomeric [SiO_4_^4−^] and [AlO_4_^4−^] formed a high polymerizate geopolymer under the action of [SiO_4_^4−^] monomer existing in waterglass. In addition, according to Table 1, it can be seen that CaO enriched in slag reacted with active SiO_2_ and Al_2_O_3_, which had not been involved in the polymerization reaction, forming amorphous C-(A)-S-H gel in the later stage of the hydration reaction of the geopolymer [33].

#### 3.4.2. Effect of Waterglass Content on the Hydration Mechanism of Geopolymer

The dissolution stage should be an exothermic process, due to the high reaction speed, and yet this was not captured in the curves of hydration temperature and cumulative heat in Figure 5 and Figure 6. Moreover, the higher the alkalinity of the geopolymer system, the faster it reacts. The waterglass content affected the quantity of OH^−^ and [SiO_4_^4−^] monomers contained in the fresh mixture; its increase accelerated both stages of the geo-polymerization stage when the modulus of waterglass was constant. Moreover, some of the [SiO_4_^4−^] uninvolved in the polymerization took part in the later hydration process, which further enhanced the densification and mechanical properties of the geopolymer.

When the waterglass content was too low, the alkalinity of the fresh mixture was not sufficient for the occurrence of the dissolution reaction described by Equations (3) and (4). As a result, the hydration and hardening of the geopolymer with 5 wt% waterglass was dependent on the alkali excitation reaction of slag and fly ash, so the exothermic peak appeared after hydrating for 72 h, resulting in the mechanical properties of the geopolymer mortar cured for 7 days accordingly. For slag-fly ash-based geopolymers, it is recommended that the waterglass content should be no less than 10 wt% to ensure a normal polymerization reaction and appropriate mechanical properties.

#### 3.4.3. Effect of Waterglass Modulus on the Hydration Mechanism of Geopolymer

Waterglass has a dual electron structure: the inner layer has amorphous SiO_2_ as the core and adsorbed silica anions on its surface, while the outer layer is a diffusion colloidal layer that determines the stability of the waterglass [34,35]. In the geo-polymerization process, the water in waterglass solution is absorbed by fine particles such as slag and fly ash or other active silicon and aluminum precursors. The thickness of the diffusion colloidal layer decreases, leading to a loss in stability and solidification and then formation of the network skeleton structure [36]. The adjustment of the waterglass modulus was realized by adding NaOH, which increased the thickness of the diffusion layer by raising the concentration of Na^+^ in the solution.

The essence of adjusting waterglass modulus is to change the alkalinity of the fresh mixture. The decrease in waterglass modulus increased the content of free Na^+^ and OH^−^ in the solution, and extended the diffusion layer, which was better for the geo-polymerization reaction. When the waterglass modulus was too low, the dissolution effect on the active precursor was enhanced, and the hydration products generated in the early stage covered the surface of the unhydrated particles, which hindered the process of the secondary hydration reaction in the later hardening stage [37]. This is demonstrated by the curves of hydration temperature and hydration heat shown in Figure 7 and Figure 8.

When the waterglass modulus was higher, the reactive silica-aluminate dissolved slowly, and the mixture became viscous which affected the leaching and migrating of oligomeric silica–alumina tetrahedra, thus, the “dissolution-polycondensation” reaction proceeded slowly. Nevertheless, the introduction of oligomeric [SiO_4_^4−^] was conducive to secondary hydration, optimizing the later mechanical properties of the geopolymer to some extent, which was initially verified by the experimental results shown in Figure 4. Therefore, the selection of reasonable intervals of waterglass modulus was crucial to ensure a normal geopolymer reaction in the early hydration stage. For slag-fly ash-based geopolymer, the waterglass modulus should be between 1.2 and 1.4.

## 4. Conclusions

This study explored the influences of waterglass content and modulus on the mechanical properties and hydration process of a slag-fly ash-based geopolymer. Through the analysis of the hydration products and their morphology, the evolution mechanism of the macroscopic properties of the geopolymer with varying waterglass content and modulus were revealed. Combined with theoretical analysis, the hydration mechanism of slag-fly ash-based geopolymer was illustrated. The main conclusions were drawn as follows:

(1) Waterglass content and modulus produced a significant effect on the mechanical properties of a slag-fly ash-based geopolymer mortar. The compressive strength of the geopolymer mortar increased with an increase in waterglass content, and first rose and then fell as the waterglass modulus increased, while its flexural strength showed a tendency to increase and then decrease with the increase in both. When the content of waterglass was 20 wt% and its modulus was 1.2, the compressive and flexural strength of the geopolymer mortar cured for 28 days were 88.4 MPa and 9.0 MPa, respectively. It is recommended that the waterglass modulus be between 1.2 and 1.4, and its content was no less than 10 wt% to ensure a suitable mechanical performance.
(2) The appropriate content and modulus of waterglass were conducive to normal geo-polymerization progress of the geopolymer mortar. The hydration temperature and cumulative hydration heat of geopolymer mortar increased with the increase in waterglass content, and decreased with the rising waterglass modulus. The time to the peak hydration temperature first shortened and then lengthened with the increase in waterglass content, while the opposite trend occurred with the change in waterglass modulus. When the geopolymer was with 20 wt% waterglass at 1.4-modulus, the hydration temperature reached its peak of 32.6 °C in 17.6 h, and the cumulative hydration heat released in 12.0 h accounted for 59.8% of its total.
(3) The hydration products of the geopolymer consisted of dense amorphous and flocculent structures wrapped around each other, and the relative amounts of both were influenced by the content and modulus of waterglass. The microstructure of the geopolymer cured for 3 days was loose when the waterglass content was 5 wt%, while hardened geopolymer cured for 3 days and 28 days was dominated by dense amorphous structures as the waterglass content increased. When the waterglass modulus was greater than 1.4, the relative areas of flocculation in the geopolymer cured for 28 days increased, forming an interface with the dense amorphous structure generated during the early hydration stage, resulting in a decrease in the mechanical properties of the geopolymer mortar.
(4) The peak temperature and time of hydration heat release of geopolymer repair mortar with various moduli and contents of waterglass was clarified, which provides a referenceable time period for pouring and using geopolymer repair mortar during its application. Based on this, the future work will focus on the deterioration law of geopolymers’ microstructure and the mechanical properties in alkaline environments to promote its utilization in practical engineering.

## Figures and Tables

**Table 1:** Chemical composition of fly ash and slag (wt%).

	SiO_2_	Al_2_O_3_	CaO	MgO	Fe_2_O_3_	SO_3_	K_2_O	Na_2_O	TiO_2_	Others
Fly ash	49.48	37.67	2.23	0.51	4.14	1.28	1.07	0.33	2.09	1.20
Slag	29.60	12.85	44.26	7.39	0.62	2.02	0.47	0.44	1.45	0.90

**Table 2:** Mixture proportion constituents of the geopolymer mortar.

Mixture ID	Geopolymer Cement (wt%)			Waterglass Modulus	W/B	B/S
	Slag	Fly Ash	Waterglass			
GC-1	66.5	28.5	5.0	1.4	0.34	0.9
GC-2	63.0	27.0	10.0	1.4	0.34	0.9
GC-3	59.5	25.5	15.0	1.4	0.34	0.9
GC-4	56.0	24.0	20.0	1.4	0.34	0.9
GC-5	56.0	24.0	20.0	1.0	0.34	0.9
GC-6	56.0	24.0	20.0	1.2	0.34	0.9
GC-7	56.0	24.0	20.0	1.6	0.34	0.9
GC-8	56.0	24.0	20.0	1.8	0.34	0.9

**Table 3:** Modulus adjustment method and results of waterglass (wt%).

Waterglass Solution	NaOH	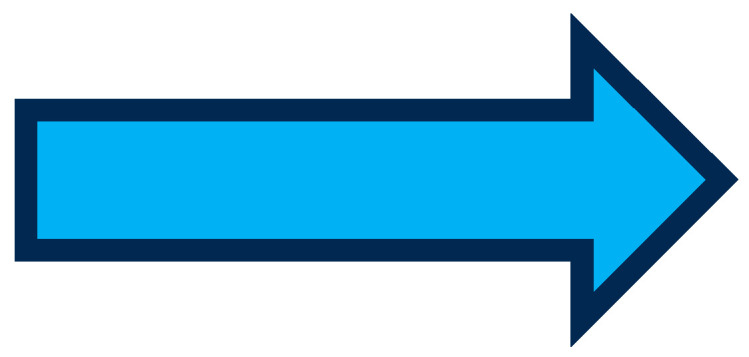	Adjusted Modulus	Solid Waterglass	Water
80.9	19.1	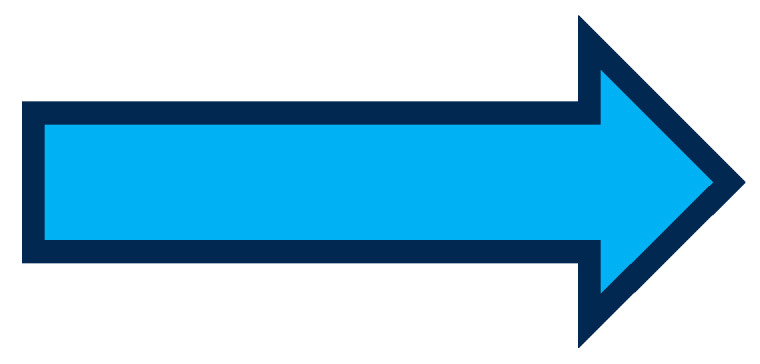	1.0	42.3	57.7
84.9	15.1		1.2	40.6	59.4
87.9	12.1		1.4	39.3	60.7
90.3	9.7		1.6	38.2	61.8
92.3	7.7		1.8	37.3	62.7

## Data Availability

The original contributions presented in this study are included in the article. Further inquiries can be directed to the corresponding authors.
